# Effects of Rhodiola Rosea Supplementation on Exercise and Sport: A Systematic Review

**DOI:** 10.3389/fnut.2022.856287

**Published:** 2022-04-07

**Authors:** Yao Lu, Bin Deng, Luhua Xu, Hanjiao Liu, Yinzhi Song, Fengxia Lin

**Affiliations:** ^1^School of Nursing, Fujian University of Traditional Chinese Medicine, Fuzhou, China; ^2^Department of Cardiology, Shenzhen Bao'an District Traditional Chinese Medicine Hospital, The Affiliated Hospital of Guangzhou University of Chinese Medicine, Shenzhen, China; ^3^Department of Nursing, Shenzhen Bao'an District Traditional Chinese Medicine Hospital, The Affiliated Hospital of Guangzhou University of Chinese Medicine, Shenzhen, China

**Keywords:** Rhodiola rosea, exercise, nutritional supplements, performance, A systematic review

## Abstract

Rhodiola rosea (Golden Root Extract; RR) is an herbaceous perennial, which is native to high altitude areas, such as East Asia, Central Asia, Siberia, and North America. It has been studied for its positive pharmacological effects on health. However, only a handful of studies have evaluated the effects of RR as an exercise supplement for sport and physical activity. The aim of this study was to evaluate whether Rhodiola can be used as a supplement to improve human exercise ability. Studies were reviewed in accordance with the PRISMA guidelines and conducted between August and November, 2021. Databases searched included Cochrane, Embase, Web of Science, PubMed and East View Universal Database. Related terms were combined with keywords and MeSH subject headings using the corresponding Boolean operators: Rhodiola rosea, arctic root, roseroot, golden root, hongjingtian, and sports and exercise. A total of 10 papers were reviewed. Most of the studies reported that RR supplementation has a positive effect on athletic ability and sports performance, and no obvious adverse reactions were reported. Subjects taking RR showed a reduction in pain and muscle damage after exercise training, improved skeletal muscle damage, enhanced antioxidant capacity thereby reducing oxidative stress, reduced RPE scores, and improved athletic explosive power, but did not reduce the rating of perceived exertion (RPE) scores. RR appears to act as a safe and effective supplementation for sport and exercise.

## Introduction

Rhodiola rosea (RR) is a perennial herb of the genus Rhodiola in the Crassulaceae family, with plants usually ranging between 10 and 30 cm in height (1). RR grows in harsh environments, mainly in rock crevices or bushes at high altitudes in East Asia, Central Asia, Siberia, and North America. There are more than 90 varieties of Rhodiola rosea. Different varieties of RR have different uses and values due to the type and content of active ingredients they contain.

More recently, RR has received attention as supplement due to its potential energy-replenishing effect, which helps to improve the physical performance. The use of RR extract by professional athletes increases physical performance and endurance, and stimulates anabolic processes in skeletal muscles. The main components that determine the phytochemical and pharmacological properties of RR are Rhodiola glycosides, benzophenones, caffeic acid, protocatechuic acid, gallic acid, and epigallocatechin gallate, amongst others. All these compounds have different biological properties (2).

In addition, previous studies have revealed valuable positive data from RR supplementation in sport and exercise performance with animal models (3). Ya Hou et al. (4) demonstrated that supplementation with RR in mice for 2 weeks ameliorates exhaustive exercise-induced fatigue, and may be associated with increased antioxidant capacity, enhanced energy production, and inhibition of mitochondrial phagocytosis in skeletal muscle.

However, the effects of RR as a supplement for human sport and physical activity have not been critically evaluated. Therefore, this review assesses the potential effects of RR as a herbal supplement for sport and exercise in humans based on the available evidence.

## Methods

### Sources and Search Strategy

The recommendations of PRISMA guidelines were followed and the research was completed in November 2021 (5). The following databases were searched: Cochrane, Embase, Web of science, PubMed and East View Universal Database between 1990 and November 2021. The search strategy involved retrieving keywords and Medical Subject Headings (MeSH) related to RR and sports, as well as combining them with the corresponding Boolean operators: “Rhodiola rosea, arctic root, roseroot, golden root, hongjingtian, sports, and exercise.” No language restrictions on search scope.

### Criteria for Study Identification and Selection

**Figure 1:**
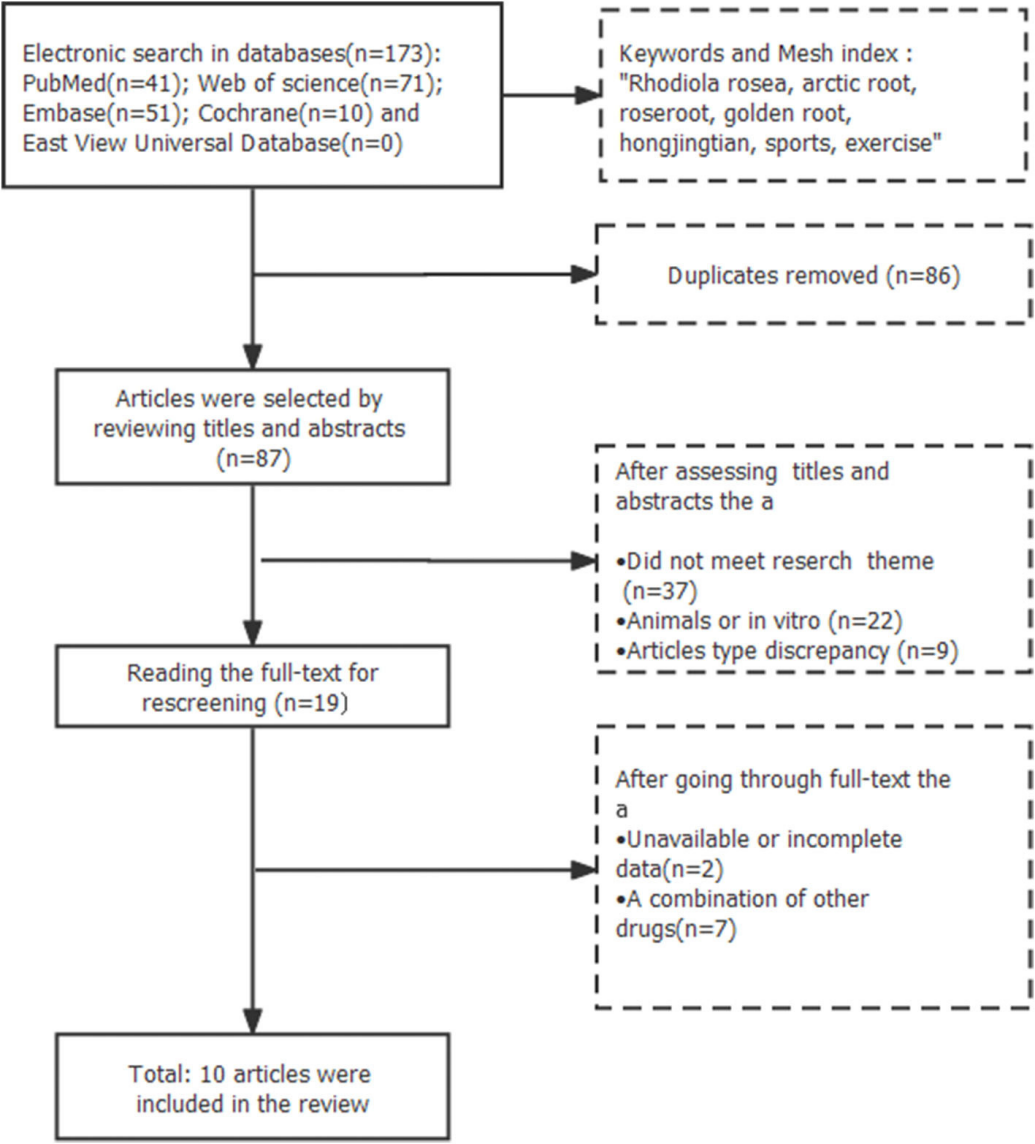
Literature screening process.

Two authors identified and selected original studies based on the eligibility criteria, and conducted an in-depth literature review of studies that might meet the requirements (Figure 1). Two authors (Lu Yao and Lin Fengxia) analyzed and selected all the papers manually, independently, and simultaneously. Disagreement was resolved through joint assessment of the literature or by referring the controversial literature to a third person for decision until consensus was reached. Through electronic retrieval, 173 papers were identified. A total of 86 duplicate publications were excluded, which was rechecked by EndNote bibliography software. The remaining 87 papers were screened after reading their titles and abstracts, as well as the full text. Finally, after a comprehensive assessment of the eligibility criteria, 10 studies entered the next stage of review.

### Eligibility Criteria

For inclusion, studies were required to be published interventional studies with human subjects who were evaluated for the effects of RR as herbal supplements on exercise and sports performance.

Studies were excluded if they were unrelated to RR supplementation, used other animals or cells as research objects, studies type discrepancy (reviews, theses, systematic reviews, meta-analyses, books, case reports, conference papers, and other non-clinical controlled research literature), were combined with other supplements or interventions, if it was not possible to obtain the full text or the data were incomplete.

### Information Extraction

Researchers recorded the author, year of publication, sample size, population characteristics, study design, intervention (including dosage and duration), source of Rhodiola Rosea extract, exercise mode, outcome variables, and main findings from each paper, in an Excel spreadsheet.

### Risk of Bias Within Studies

Two researchers worked independently to evaluate the risk of bias used the Cochrane ROB's tool. The following aspects were included: random sequence generation, allocation concealment, blinding of investigators and subjects, blinded evaluation of study outcomes, completeness of outcome data, selective reporting of study results, and other sources of bias. Each of the above entries was judged as “low risk of bias, unclear, or high risk of bias.”

### Data Analysis

We converted outcomes to standard units and calculated mean difference. Differences in related indexes between the Rhodiola rosea treatment group and the placebo treatment group were analyzed using a factorial (time × treatment) ANOVA comparison.

## Results

### Selection and Description of the Studies

We identified 173 potentially relevant articles from five databases. After removal of duplicates and irrelevant articles, 87 records remained. By reviewing titles and abstracts, 68 studies were excluded because the following reasons: (1) the article did not meet research theme; (2) the researches were animals or *in vivo* study; (3) they were case reports, abstracts, comments, clinical trials, editorials, letters and review articles. After going through the remaining full-text articles, nine articles were excluded because of having unavailable or incomplete data or a combination of other drugs. Finally, 10 studies that met the inclusion criteria were included in this review (Figure 1). In these studies, the subjects consisted of healthy adults with sample sizes ranging from 10 to 48, mostly randomized and crossover clinical trials, with intervention cycles divided into short- and long-term interventions, ranging from 1 h to 37 days. Five studies examined the effects of short-term RR on exercise performance, and five assessed the effects of RR as a long-term supplement. The types of exercise tests included aerobic, anaerobic, resistance, and endurance (Table 1).

**Table 1:** Characteristics of the studies include.

**References**	**Study design**	**Sample**	**Age**	**Intervention/ control**	**Duration**	**RR extract**	**Exercise test**
Williams et al. (6)	Randomized double-blinded, crossover, counterbalanced clinical trial	10 Resistance-trained individuals (10 M)	24.8 ± 5.6 years	Group1: 1,500 mg/day of Rhodiola rosea and took an additional 500 mg dose of corresponding treatment 30 min perid to exercise testing. Group2: placebo (PL; gluten-freecornstarch)	3-day + 30 min prior	NOW Food Inc., Bloomingdale, IL, USA	Resistance exercise: 1set × 2 explosive reps at 75% of one-repetition maximum(1 RM), 3 sets × repetitions to failure (RTF) at 75% 1 RM
MedBallmann et al. (7)	Randomized blinded and counter- balanced clinical trial	11 Physically active participants (11 W)	19.4 ± 0.8 years	Group1: Supplemented with 500 mg of Rhodiola rosea three times daily (~1,500 mg/day)and took an additional 500 mg dose of corresponding treatment 30 min prior to testing of each trial. Group2: Placebo (glu- ten-free cornstarch)	3-day+30 min prior	NOW Food Inc., Bloomingdale, IL, USA	Completed 3 × 15 second Wingate Anaerobic Tests (WAnTs)
Lin et al. (8)	Randomized double-blind, crossover clinical trial	12 heathy subjects. (12 M)	24.7 ± 0.5 years	Group1: Two Rhodiola rosea capsules (400 mg Rhodiola rosea per capsule) daily. Group2: Placebo (hydrox- ymethylcellulose)	After 3-day exercise period and during 5-day recovery period	Standard Chem & Pharm Co., Taiwan	Continuous endurance exercise: 30-min run at 75% VO2max
Jowko et al. (9)	Randomized double-blind clinical trial	26 healthy students. (26 M)	PL: 20.9 ± 0.2 years. RR: 20.5 ± 0.3 years	Group1: 600 mg/day Rhodiola rosea Group2: Placebo	4 weeks before exercise test	Naturell, Sweden	Incremental cycle ergometer tests
Duncan et al. (10)	Double-blind crossover clinical trial	10 healthy young individuals and recreation exercisers. (10 M)	26 ± 6 years	Group1: 3 mg/kg body mass of Rhodiola rosea placed in a colored, opaque gelatin capsule. Group2: Placebo	60 min before exercise test	Indigo Herbs, Glastonbury, UK	Resistance exercise: completed two 30-min submaximal cycling trials at a workload of 70% VO_2maxi_n a fasted state
Shanely et al. (11)	Randomized double-blind clinical trial	48 runners. (35 M and 13 W)	25–65 years	Group1: 600 mg/day Rhodiola rosea extract before running a marathon (2 Rhodiola rosea capsule and each capsule contained 300 mg of Rhodiola rosea extract). Group2: Placebo	30 days prior to and the day of the marathon and seven days after marathon	PL Thomas & Co., Inc. (Morristown New Jersey)	A competitive marathon
Noreen et al. (12)	Randomized double-blind,crossover clinical trial	18 recreationally active subjects. (18 W)	22 ± 3.3years	Group1: 3 mg/kg of Rhodiola rosea. Group2: Placebo (carbohydrate)	1 h before test	Bulknutrition.com, Northborough, MA, USA	10-min warm-up followed by a simulated 6-mile time trial on a variable grade course using the Velotron electronic bicycle ergometer
Parisi et al. (13)	Double-blind clinical trial	14 well-trained athletes. (14 M)	25 ± 5 years	Group1: 170 mg/day Rhodiola rosea. Group2: Placebo	4 weeks before exercise test	Unspecified	Cardio-pulmonary exhaustion test with the cycloergometer at 75% VO2max
Skarpanska- Stejnborn et al. (14)	Randomized double-blind clinical trial	22 professional rowers. (22 M)	PL: 21.0 ± 0.9 years. RR: 20.4 ± 1.2 years	Group1: 100 mg of Rhodiola rosea extract twice daily. Group2: Placebo	4 weeks before exercise test	Unspecified	2,000-m maximum test on a rowing ergometer
Walker et al. (15)	Randomized double-blind clinical trial	12 resistance -trained subjects. (12 M)	29.92 ± 4.51 years	Group1: 1,500 mg/day Rhodiola rosea and 1,000 mg the day of the test. Group 2: Placebo	3-day + the day of the test	Bali Herbal, Singapore	Incremental forearm wrist flexion exercise to volitional fatigue

Additional details about the papers, including population characteristics and dosage of RR and placebo, are described in Tables 1, 2 exhibits the details about the outcome variables (pain and muscle damage, inflammation, rating of perceived exertion, antioxidant capacity, skeletal muscle damage, and athletic explosive power), in addition to the description of the conclusions reported in the papers.

**Table 2:** Outcome variables and main results.

**References**	**Outcome variables**	**Main results**
Williams et al. (6)	pre-(PRE), immediately post-(POST) exercise:LA, EPI, NE. Mean barbell velocity and total volume performed	Mean concentric velocity was significantly higher with GRE compared to PL (*p* = 0.046). Total RTF were significantly lower with GRE versus PL (*p* < 0.001). GRE resulted in greater Post values compared to PL (*p* = 0.049). EPI and NE increased in both conditions Pre to Post (*p* < 0.001). However, Pre NE was significantly higher with GRE versus PL (*p* = 0.008)
MedBallmann et al. (7)	Mean watts, mean anaerobic capacity, mean anaerobic power, mean peak watts, mean total work, mean fatigue index	Mean watts (*p* = 0.017, ES = 0.55), mean anaerobic capacity (*p* = 0.025, ES = 0.96),mean anaerobic power (*p* = 0.03, ES = 1.07),mean peak watts (*p* = 0.029,ES=0.46),and mean total work (*p* = 0.017, ES = 0.49)were higher in the GRE treatment trial versus placebo. However, mean fatigue index (*p* = 0.094, ES = 0.39) was unaffected regardless of treatment
Lin et al. (8)	before,immediately after,and1,2,24,48,72,and 120 h after :CK,LA, IL-1beta,IL-6, TNF-α, and CRP	At 24 and 48 h after the exercise,CK levels were higher than those before exercise in both groups.T he blood variables had returned to the baseline level at 24 h after exercise except the CK level. The CK levels were lower in the group 1 than that in the group 2 72 h after continuous exercise(208.2 ± 32.7 vs.136.7 ± 13.8U/L, *P* < 0.05). The supplementation of Rhodiola rosea after continuous endurance exercise has a trend to decrease blood CK levels moderately, but it is unable to reduce inflammatory reactions
Jowko et al. (9)	VO_2_ peak, TTE, PLT, and LAmax, HR values, Pmax, LArest	VO_2_ peak, TTE, PLT, and LAmax, as well as HR values did not change significantly in either group. Significant decrease in peak power (Pmax) in PL group in TermII as compared to TermI (*p* < 0.05), In RR group, Pmax did not change. Resting LA concentration was significant lower in RR group when compared to PL group (*p* < 0.05)
Duncan et al. (10)	Rest, 10, 20, and 30 min into the exercise: Heart rate; RPE; BRUMS; pleasure after exercise; rate of total carbohydrate oxidation	RPE was lower at 30 min into exercise for group 1 versus placebo (*p* = 0.003). Perceptions of arousal (*p* = 0.05) and pleasure were higher after exercise for group1 compared to placebo. Mood state scores for vigor were higher in group 1 compared to placebo (*p* = 0.008). Ingestion of R.rosea did not result in significant differences in energy expenditure, carbohydrate, or fat oxidation compared to placebo (*p* > 0.05)
Shanely et al. (11)	The day before,15 min post-and 1.5 h post-marathon: vertical jump and DOMS. Blood sample:Mb, CPK, AST, ALT, IL-6, IL-8, IL-10, MCP-1, G-CSF, CRP, eHSP72	Intake RR supplementation did not attenuate the post-marathon decreased in muscle function, or increases in muscle damage, DOMS, eHSP72, or plasma cytokines in experienced runners, and the magnitude of change did not differ between groups
Noreen et al. (12)	RPE, Blood lactate concentration, salivary cortisol, salivary alpha amylase, heart rate, time to completion, average power	During the standardized warm-up the average heart rate (*p* = 0.001)and the PRE during the time trial (*p* = 0.04) in RR group was lower when compared to PL group. Time to completion during the time trial in RR group was lower when compared to PL group (*p* = 0.037). No significant differences between treatments for average power, average heart rate, average cadence, salivary, hormones, blood lactate
Parisi et al. (13)	HR Max, Borg Scale level, VO_2_max, TAS, CK, GLU, FFAs, MDA	Blood antioxidant status,inflammatory parameters, blood glucose,HR Max,Borg Scale level,VO_2_max and duration of the test were essentially unaffected by RR but plasma free fatty acids levels were reduced. Blood lactate and plasmacreatine kinase levels were found significantly lower (*P* < 0.05) in RR treated subjects when compared to the placebo
Skarpanska-Stejnborn et al. (14)	Power Output, blood lactate (La) Levels, total run time, SOD, GPx, UA, TAC, CK, TBARS	The total plasma antioxidant capacity was significantly higher (*p* = 0.0002) in RR group than in the PL group, and superoxide dismutase activity in erythrocytes directly after and 24 h after the ergometry was significantly (*p* = 0.0461) lower in athletes receiving RR extracts than in the PL group
Walker et al. (15)	During exercise and recovery from exercise: PC, rating of perceived exertion per stage, time to exhaustion	There were no significant differences between groups for PC, rating of perceived exertion per stage, time to exhaustion. RR ingestion does not improve ATP turnover during or immediately after exercise

### Rhodiola Rosea Extraction and Standards

Rhodiola rosea used in the included studies was mostly commercially available, are described in Table 1. The extraction methods as well as the quantification criteria differed significantly from one manufacturer to another. The Rhodiola rosea supplement was standardized to a minimum of 3 total Rosavins and 1% total Salidroside (6–9, 11, 12, 15). However, some studies did not show the content of rosavins and salidroside in Rhodiola rosea (10, 13, 14).

### Pain and Muscle Damage

Compared with the placebo group, there was a tendency for blood creatine kinase (CK) levels to decrease in the Rhodiola group 72 h after continuous exercise (208.2 ± 32.7 vs. 136.7 ± 13.8 U/L, *P* < 0.05) (8). In another study, the CK levels showed a significant reduction for the RR group in comparison with the placebo group in rest time (19.35 ± 2.96 vs. 34.26 ± 5.95 U/L, *P* < 0.05), acme (23.50 ± 3.96 vs. 37.19 ± 7.29 U/L, *P* < 0.05) and 30 min recovery time samples (25.70 ± 5.24 vs. 35.52 ± 4.20 U/L, *P* < 0.05)_._ There was also a significant improvement in skeletal muscle damage after supplementation with RR supplement (13). Observed that chronic RR supplementation (170 mg/day RR for 4 weeks) reduced skeletal muscle injury parameters after exhaustive exercise. Another study (7) reported that RR extract can lower LDH levels in blood serum and protect skeletal muscle cells. However, another clinical trial with 48 professional marathon runners supplemented with RR extract (600 mg/day) for 30 days before a marathon competition, found no significant effects on muscle function, pain, or muscle damage, delayed onset muscle soreness without statistical difference in the RR and placebo control groups (*p* = 0.70) (11)_._

Phosphocreatine (PCr) kinetics is a reflection of adenosine triphosphate (ATP) synthesis. Although rating of perceived exertion significantly increased within groups as workload increased, it did not differ in time to exhaustion (10.71 ± 0.54 vs. 10.48 ± 0.68 min, *P* < 0.05). Estimates of PCr at time 0, 5, 10, 15, and 20 min of recovery were nearly identical between groups (15).

### Inflammation

RR is a medicinal plant with anti-inflammatory properties. It is believed that RR can reduce various stressors caused by chemical and biological factors, however, this effects is not related to inflammatory cytokines. A study (8) with 12 heathy subjects found that serum levels of inflammatory indicators, such as IL-1β, IL-6, TNF-α, and CRP, were not reduced in the RR group (400 mg/day, 8 days) compared with the placebo group after continuous endurance exercise (30-min run at 75% VO_2max_). Another study (11), with 48 runners competing in a marathon, reported that total blood leukocyte counts, IL-6, IL-8, IL-10, MCP-1, G-CSF, CRP, and eHSP72 all increased significantly, however, no significant differences were found for the RR group (600 mg/day, 30 days) compared with the placebo group (*P* = 0.526).

### Rating of Perceived Exertion

The rating of perceived exertion (RPE) is a subjective measure of how one feels during exercise using the Borg 6–20 scale (16). Duncan et al. (10) concluded that ingestion of RR favorably influenced RPE and exercise effect in regularly active adults (age: 26 ± 6 years). RPE significantly decreased in the RR group (3 mg/kg) after 30 min of exercise compared with the placebo group (*P* = 0.003). Noreen et al. (12) observed improved exercise endurance performance after RR intake, with a lower mean RPE in the RR test (*P* = 0.04). This difference was more pronounced when calculating the ratio of RPE relative to workload (*P* = 0.007).

### Oxidative Stress

Regarding oxidative stress, Jowko et al. (9) observed resting plasma total antioxidant capacity (TAC) increased significantly in the RR group, whereas no significant changes were found in placebo group. In another study (14), 22 professional rowers were tested on a rowing ergometer and randomly assigned to either the RR or placebo group for 4 weeks. The results showed that the total plasma antioxidant capacity of the study subjects in the supplementation group was significantly higher than that of the placebo group (*P* = 0.0002). In contrast, Parisi et al. (13) reported plasma total antioxidant status was similar after RR supplementation when compared with placebo. According to their research, chronic long-term intake of RR did not affect plasma enzymatic and non-enzymatic antioxidant systems.

### Athletic Explosive Power

Only one paper evaluated the effect of RR supplementation on the improvement of athletic explosive power during repeated bench press exercises. In a study by Williams et al. (6), subjects were given 1,500 mg/day of RR and an additional 500 mg dose 30 min prior to exercise testing, and results indicated this may enhance the explosive power of sports compared with placebo, but at the same time, may damage upper body strength and endurance.

### Adverse Effects

Among the 183 individuals enrolled in the 10 clinical studies included, none of the doses of RR showed significant clinical side effects and no adverse effects on health damage were reported.

### Risk of Bias Assessment in RR Trials

Although most studies were randomized controls, many did not describe in detail the method of random sequence generation, which affected the assessment. The 10 studies that were included, were double blinded and therefore had a low risk of bias associated with allocation concealment. All included studies were considered to have indistinct reporting of selective outcomes due to inadequate information. In the assessment of “other risks of bias,” two studies had a high risk of bias—one study did not describe the sample size calculation method (6), and the other study had a baseline imbalance due to participants' ages (11).

## Discussion

It is shown that Rhodiola rosea as an exercise supplement has the ability to improve human exercise capacity and a variety of physiological mechanisms, improving the body's resistance to fatigue from exercise. Rhodiola rosea preparations exhibit adaptogenic effects, including neuroprotective, cardioprotective, anti-fatigue and central nervous system stimulating activity. The mechanisms of action include interactions with HPA-system (cortisol-reducing) (17), protein kinases p-JNK (18), nitric oxide (19), and defense mechanism proteins.

The majority of RR used in the above mentioned studies are commercially available or available from other pharmacy facilities. Firstly the location and timing of harvest, the methods of extraction, and the presence or absence of adulterants vary considerably among RR products. Secondly, because the dose and timing of administration significantly affect the results, it is necessary to use high-performance liquid chromatography for the quality control of RR. Studies of synthetic pharmaceuticals are easier to evaluate because only one substance is investigated. In contrast, studies of herbs are more challenging because highly complex herbs (e.g., RR) contain dozens of bioactive constituents with polyvalent effects (20). Marker compounds are insufficient to assure purity because they can now be synthesized and added to increase their concentration to meet standardization requirements. liquid chromatography-mass spectrometry pseudotargeted analysis produces a profile of constituents that helps with identification (21). For that matter, DNA testing of root stock used to produce products would help assure purity (22).

This review demonstrates clearly that RR supplementation has the potential to improve sport and exercise performance through a reduction in oxidative stress, muscle pain and injury, improved skeletal muscle damage and muscle recovery during training, as well as an increase in athletic explosive power.

Researchers in the USSR have demonstrated that Rhodiola rosea can prolong muscle loading time by promoting protein hydrolysis, thus reducing muscle soreness or muscle injury after exercise. Research evidence suggests that post-exercise muscle damage is associated with increased concentrations of CK and CRP in the blood following high-intensity exercise (23). After RR intake, however, the elevation of blood lactate levels was significantly reduced and CK levels were also significantly lower in participants. It is therefore hypothesized that chronic RR supplementation may not only reduce exercise-induced muscle damage, but may also have a preventive effect. The mechanism may be that RR improves exercise capacity and protects against muscle damage by enhancing mitochondrial quality control, including the activation of mitophagy, mitochondrial dynamics, and biogenesis (3).

Numerous experimental studies in animals and subjects have shown that skeletal muscle atrophy is associated with the expression of several inflammatory cytokines (24, 25). RR glycosides can exert their anti-inflammatory effects by acting on the relevant inflammatory cytokines (26). The anti-inflammatory effect of RR is mediated by its action on the relevant inflammatory cytokines, thereby alleviating the atrophy of skeletal muscle (27). However, there are different theories on the anti-inflammatory effects of RR on physical activity, with some concluding that RR supplementation did not significantly reduce the expression of inflammatory factors (8, 11).

Strenuous exercise leads to the production of free radicals and other reactive oxygen/nitrogen species in muscle tissue (28). In addition, the levels of oxidative stress-responsive substances in skeletal muscle play a key role in regulating muscle function (29). Recent research, suggests that RR extract can increase antioxidant capacity by upregulating antioxidant enzymes, reduce skeletal muscle damage, and increase the body's resistance to fatigue (30). Supplementation with different types of antioxidants, including RR, has been recognized as a positive strategy to prevent oxidative stress and improve athletic performance (14).

Animal experiments have shown that RR increases adenosine triphosphate (ATP) and creatine phosphate. RR-treated rats significantly prolonged the duration of exhaustive swimming, and RR had the effect of activating the synthesis or resynthesis of ATP in mitochondria and promoting the process of energy recovery in the body after exhaustive exercise (31). RR enhances cell regeneration and energy metabolism by increasing the synthesis of adenosine triphosphate, ribonucleic acid, protein, and amino acid (32).

The data from the studies in this review indicate that there were no obvious adverse reactions from RR, its herbal toxicity is very low, and the test doses appear to be safe and tolerable for recreational active subjects or athletes.

## Conclusions

There are some limitations in this study. Firstly, it was not possible to perform a meta-analysis of their data because the included studies showed relatively obvious heterogeneity. Secondly, the methodological quality of the included original studies was generally low, and most studies did not elaborate on the specific methods of implementation of allocation concealment and blinding, which can limit the credibility of the findings. Thirdly, the sample size of most of the included literature was not adequate, which limits the reliability of the results. Considering these limitations, more rigorous randomized controlled trials with adequate sample sizes are needed to replicate and validate the results of published randomized controlled trials. As stated in the IOC consensus statement (33), both RR and other sports supplements are urgently needed for randomized controlled trials in high-level athletes with adequate numbers of participants, rigorous controls and procedures, appropriate supplementation protocols, and clinically meaningful measurement test.

Despite its limitations, this systematic review provide valuable information for the application of RR supplementation in improving sport and exercise performance. RR has the potential to reduce oxidative stress, muscle pain and injury, improve skeletal muscle damage and muscle recovery during training, as well as improve athletic explosive power. With the gradual improvement in quality of life and the increasing demand for sports and wellness drugs, RR is expected to become an effective supplement.

## Data Availability Statement

The original contributions presented in the study are included in the article/supplementary material, further inquiries can be directed to the corresponding author.

## Author Contributions

FL and YL designed and planned the study and identified and selected original studies. YL drafted the manuscript. FL and YS revised the final manuscript. BD and LX contributed to data analysis. FL and HL provided advice during the study and manuscript preparation. All authors read and approved the final manuscript.

## Funding

This study was supported by the National Natural Science Foundation of China (82004320) and the Science and Technology Project of Shenzhen City of China (JCYJ20190807115201653).

## Conflict of Interest

The authors declare that the research was conducted in the absence of any commercial or financial relationships that could be construed as a potential conflict of interest.

## Publisher's Note

All claims expressed in this article are solely those of the authors and do not necessarily represent those of their affiliated organizations, or those of the publisher, the editors and the reviewers. Any product that may be evaluated in this article, or claim that may be made by its manufacturer, is not guaranteed or endorsed by the publisher.
