# Corrigendum: Effects of Rhodiola Rosea Supplementation on Exercise and Sport: A Systematic Review

**DOI:** 10.3389/fnut.2022.928909

**Published:** 2022-06-20

**Authors:** Yao Lu, Bin Deng, Luhua Xu, Hanjiao Liu, Yinzhi Song, Fengxia Lin

**Affiliations:** ^1^School of Nursing, Fujian University of Traditional Chinese Medicine, Fuzhou, China; ^2^Department of Cardiology, Shenzhen Bao'an District Traditional Chinese Medicine Hospital, The Affiliated Hospital of Guangzhou University of Chinese Medicine, Shenzhen, China; ^3^Department of Nursing, Shenzhen Bao'an District Traditional Chinese Medicine Hospital, The Affiliated Hospital of Guangzhou University of Chinese Medicine, Shenzhen, China

**Keywords:** Rhodiola rosea, exercise, nutritional supplements, performance, A systematic review

In the original article, there was a mistake in Table 1 as published. Some of the reference details in Table 1 were not complete. The corrected Table 1 appears below.

**Table 1:** Characteristics of the studies include.

**References**	**Study design**	**Sample**	**Age**	**Intervention/** **control**	**Duration**	**RR extract**	**Exercise test**
Williams et al. (6)	Randomized double-blinded, crossover, counterbalanced clinical trial	10 Resistance-trained individuals (10 M)	24.8 ± 5.6 years	Group1: 1,500 mg/day of Rhodiola rosea and took an additional 500 mg dose of corresponding treatment 30 min perid to exercise testing. Group2: placebo (PL; gluten-freecornstarch)	3-day + 30 min prior	NOW Food Inc., Bloomingdale, IL, USA	Resistance exercise: 1set × 2 explosive reps at 75% of one-repetition maximum(1 RM), 3 sets × repetitions to failure (RTF) at 75% 1 RM
MedBallmann et al. (7)	Randomized blinded and counter- balanced clinical trial	11 Physically active participants (11 W)	19.4 ± 0.8 years	Group1: Supplemented with 500 mg of Rhodiola rosea three times daily (~1,500 mg/day)and took an additional 500 mg dose of corresponding treatment 30 min prior to testing of each trial. Group2: Placebo (glu- ten-free cornstarch)	3-day+30 min prior	NOW Food Inc., Bloomingdale, IL, USA	Completed 3 × 15 second Wingate Anaerobic Tests (WAnTs)
Lin et al. (8)	Randomized double-blind, crossover clinical trial	12 heathy subjects. (12 M)	24.7 ± 0.5 years	Group1: Two Rhodiola rosea capsules (400 mg Rhodiola rosea per capsule) daily. Group2: Placebo (hydrox- ymethylcellulose)	After 3-day exercise period and during 5-day recovery period	Standard Chem & Pharm Co., Taiwan	Continuous endurance exercise: 30-min run at 75% VO2max
Jowko et al. (9)	Randomized double-blind clinical trial	26 healthy students. (26 M)	PL: 20.9 ± 0.2 years. RR: 20.5 ± 0.3 years	Group1: 600 mg/day Rhodiola rosea Group2: Placebo	4 weeks before exercise test	Naturell, Sweden	Incremental cycle ergometer tests
Duncan et al. (10)	Double-blind crossover clinical trial	10 healthy young individuals and recreation exercisers. (10 M)	26 ± 6 years	Group1: 3 mg/kg body mass of Rhodiola rosea placed in a colored, opaque gelatin capsule. Group2: Placebo	60 min before exercise test	Indigo Herbs, Glastonbury, UK	Resistance exercise: completed two 30-min submaximal cycling trials at a workload of 70% VO_2maxi_n a fasted state
Shanely et al. (11)	Randomized double-blind clinical trial	48 runners. (35 M and 13 W)	25–65 years	Group1: 600 mg/day Rhodiola rosea extract before running a marathon (2 Rhodiola rosea capsule and each capsule contained 300 mg of Rhodiola rosea extract). Group2: Placebo	30 days prior to and the day of the marathon and seven days after marathon	PL Thomas & Co., Inc. (Morristown New Jersey)	A competitive marathon
Noreen et al. (12)	Randomized double-blind,crossover clinical trial	18 recreationally active subjects. (18 W)	22 ± 3.3years	Group1: 3 mg/kg of Rhodiola rosea. Group2: Placebo (carbohydrate)	1 h before test	Bulknutrition.com, Northborough, MA, USA	10-min warm-up followed by a simulated 6-mile time trial on a variable grade course using the Velotron electronic bicycle ergometer
Parisi et al. (13)	Double-blind clinical trial	14 well-trained athletes. (14 M)	25 ± 5 years	Group1: 170 mg/day Rhodiola rosea. Group2: Placebo	4 weeks before exercise test	Unspecified	Cardio-pulmonary exhaustion test with the cycloergometer at 75% VO2max
Skarpanska- Stejnborn et al. (14)	Randomized double-blind clinical trial	22 professional rowers. (22 M)	PL: 21.0 ± 0.9 years. RR: 20.4 ± 1.2 years	Group1: 100 mg of Rhodiola rosea extract twice daily. Group2: Placebo	4 weeks before exercise test	Unspecified	2,000-m maximum test on a rowing ergometer
Walker et al. (15)	Randomized double-blind clinical trial	12 resistance -trained subjects. (12 M)	29.92 ± 4.51 years	Group1: 1,500 mg/day Rhodiola rosea and 1,000 mg the day of the test. Group 2: Placebo	3-day + the day of the test	Bali Herbal, Singapore	Incremental forearm wrist flexion exercise to volitional fatigue

The authors apologize for this error and state that this does not change the scientific conclusions of the article in any way. The original article has been updated.

## Publisher's Note

All claims expressed in this article are solely those of the authors and do not necessarily represent those of their affiliated organizations, or those of the publisher, the editors and the reviewers. Any product that may be evaluated in this article, or claim that may be made by its manufacturer, is not guaranteed or endorsed by the publisher.

